# The impact of central sensitization on perioperative pain in TKA: a retrospective cohort study

**DOI:** 10.1186/s43019-025-00263-8

**Published:** 2025-03-19

**Authors:** Tatsuru Sonobe, Takuya Nikaido, Miho Sekiguchi, Yoichi Kaneuchi, Tadashi Kikuchi, Yoshihiro Matsumoto

**Affiliations:** 1 Department of Orthopedic Surgery, Fukushima Medical University School of Medicine, 1 Hikarigaoka, Fukushima-Shi, Fukushima 960-1295 Japan; 2 Department of Orthopedic Surgery, Bange-Kosei General Hospital, Fukushima, 969-6593 Japan

**Keywords:** Knee osteoarthritis, Central sensitization, Total knee arthroplasty, KOOS pain, Self-efficacy

## Abstract

**Background:**

Total knee arthroplasty (TKA) is an established surgical procedure for severe knee osteoarthritis (KOA) that has provided excellent outcomes. While several studies have reported that patients with preoperative central sensitization (CS) experienced worse pre- and post-operative pain and outcomes, the evidence is limited. We conducted this study to determine the impact of CS on perioperative knee pain in TKA for severe KOA.

**Methods:**

A retrospective cohort study of 66 patients who underwent bilateral TKA for bilateral severe KOA was conducted. Multiple linear regression models that included covariates and scaled estimated regression coefficients were used to examine the impact of CS on the patients’ pre- and post-operative pain subscale values on the Knee Injury and Osteoarthritis Outcome Score (KOOS) and the improvement of KOOS pain. Postoperative KOOS pain was assessed at 3 months postoperatively, while other evaluation items including preoperative KOOS pain, CS, and pain self-efficacy were assessed on admission.

**Results:**

CS had a negative impact on pre- and post-operative KOOS pain (preoperative, *β*: −0.28, 95% confidence interval [CI] −18.53, −0.92; postoperative, *β*: −0.26, 95%CI −14.09, −0.44; *p* < 0.05). High pain self-efficacy had a positive impact on preoperative KOOS pain (*β*: 0.25, 95%CI 0.32, 18.08; *p* < 0.05). However, CS did not influence the improvement of KOOS pain.

**Conclusions:**

These results demonstrate that CS had a negative impact on pre- and post-TKA knee pain in patients but did not affect the improvement of knee pain. TKA provides sufficient pain relief for severe KOA, with or without CS. Further research is required to improve pre- and post-operative knee pain in KOA patients with CS.

## Background

Total knee arthroplasty (TKA) is an established surgical procedure for severe knee osteoarthritis (KOA) that has provided excellent outcomes and patient satisfaction [1, 2]. However, despite good clinical outcomes, some TKA cases have low postoperative patient-based outcome scores [3]. Pain is the main cause of dissatisfaction for most of these patients [4]. Pain may have a psychological component, related to anxiety and depression, and/or the stress response [4]. A systematic review revealed that 8.0–26.5% of TKA recipients reported postoperative residual pain [5]. Some patients reported chronic postsurgical pain (CPSP) [4].

Central sensitization (CS) is one of the major causative factors of CPSP and has attracted attention as a therapeutic target [6]. CS is defined as an amplification of neural signals within the central nervous system that induces pain sensitivity [7]. The estimated prevalence of CS is approximately 30% of patients with OA [8]. According to a systematic review, CS is closely associated with more severe and persistent pain after TKA, and appropriate patient education regarding common postoperative pain patterns is important [9]. A few studies have reported that patients with preoperative CS experienced worse pre- and post-operative pain and outcomes [10, 11], but the number of such reports is limited. By understanding the impact of CS on perioperative knee pain in patients undergoing TKA for severe knee OA, surgeons can optimize treatment for these patients. This study aims to determine the impact of CS on perioperative knee pain in patients undergoing TKA.

## Methods

### Patients

A retrospective cohort study of 66 patients who underwent bilateral TKA for bilateral KOA at Bange-Kosei General Hospital (Fukushima, Japan) during the period from December 2022 through November 2023 was conducted. All patients with Kellgren–Lawrence grade [12] (KL grade) III or IV KOA in both knees and who underwent bilateral TKA during the inclusion period were enrolled, without age restriction. All surgeries were performed one side at a time, with the contralateral side performed 14 days after the unilateral surgery. All patients underwent the same surgical protocol and the implants used were consistent. Postoperative pain control and rehabilitation protocols were also consistent in all patients.

Patients with a history of previous knee surgery, trauma, rheumatoid arthritis, or hip pathology were excluded. Patients with cognitive decline who were unable to complete the questionnaire were also excluded. We obtained the patients’ demographics from their medical records, including age, sex, and body mass index (BMI).

### Radiographic KOA severity

The patients’ radiographic KOA severity was graded on the basis of the KL grade. In total, two well-trained knee surgeons assessed the anterior‒posterior view of both knee plane radiographs of the patient in the standing position. In this study, all patients had severe KOA (KL grade 3 or 4) in both knees, with the KL grade determined on the basis of the patient’s more severe side. When the knee surgeons’ assessment of the KL grade for a patient did not match, consensus was reached via discussion.

### The CSI-9, PSEQ, and BS-POP

The Central Sensitization Inventory-9 (CSI-9), Pain Self-Efficacy Questionnaire (PSEQ), and Brief Scale for Psychiatric Problems in Orthopedic Patients (BS-POP) were evaluated to assess patients’ psychological factors for knee pain. Each questionnaire was self-administered on admission.

The Central Sensitization Inventory (CSI) is a self-questionnaire that rates 25 health-related symptoms common to CS on a scale of 0 to 4 points [13]. The CSI-9 is a nine-item, simplified version of the CSI. The CSI-9 classifies central sensitization into three levels of severity: subclinical with a score of 0–9 points, mild with a score of 10–19 points, and moderate/severe with a score of 20–36 points [14]. In addition, even cases with a CSI classified as mild have been shown to have more severe pain and increased CS-related disease compared with those without a CS [15]. We thus defined a CSI score ≥ 10 points as “high CS” in the present study, and we classified the patients with high CS scores in the CS group (C group) and those with a score < 9 points as the non-CS group (N group).

Pain self-efficacy is a positive cognitive factor and is considered a protective factor that contributes to adaptation despite pain [16]. The PSEQ is a ten-item self-reported questionnaire designed to evaluate the degree of confidence in one’s ability to perform a variety of activities despite experiencing pain [17]. Each item of the PSEQ is rated on a seven-point Likert scale (with 0 signifying not confident at all and 6 signifying completely confident). Total scores range from 0 to 60 points, with higher scores indicating greater pain self-efficacy to perform activities even in the presence of pain. The PSEQ used in the present study was shown to be reliable on the basis of a systematic review of pain self-efficacy measures [18]. As in other reports [17, 18], a score of ≥ 40 points was defined as high pain self-efficacy in the present study.

Psychiatric problems such as anxiety and depression are associated with postoperative pain in patients who have undergone a TKA [19]. The BS-POP is a questionnaire used to assess psychiatric problems in clinical practice [20], with two components: one for physicians and one for patients. The physician version consists of eight questions, with the physician answering each question on the basis of the patient’s assessment. Each question is rated on a three-point scale, with total scores ranging from 8 to 24, with higher scores indicating more problems. The patient version of the BS-POP consists of ten questions, which the patient completes to assess mood problems. Each item is rated on the same scale as the physician version, with total scores ranging from 10 to 30 points, with higher scores indicating more severe psychiatric problems. In the present study, a score ≥ 11 points on the physician version or a combination of ≥ 10 points on the physician version and ≥ 15 points on the patient version was defined as an abnormal BS-POP result; lower scores were defined as a normal BS-POP result [20].

### Knee pain

A validated version of the Knee Injury and Osteoarthritis Outcome Score (KOOS) [21] was applied to each patient. We focused on the pain subscale among the five subscales of the KOOS in this study. Each subscale is independently rated as 0 to 100 points (0 meaning severe knee problems and 100 meaning no problems). The patients’ preoperative KOOS pain was measured at the time of admission, and their postoperative KOOS pain was evaluated at 3 months after bilateral TKA. The degree of improvement in knee pain associated with TKA surgery was defined as a patient’s KOOS pain value at 3 months postoperatively minus his/her preoperative KOOS pain value.

### Ethical consideration

Written informed consent for the use of the data collected in this study was obtained from all patients upon enrollment. The study complied with the Declaration of Helsinki and was approved by the research ethics committee of our university (no. 2022-175).

### Statistical analyses

Descriptive statistics were calculated for the patients’ baseline characteristics. Continuous data were summarized as the mean and standard deviation, and dichotomous or categorical data were presented as proportions. Comparative analyses of KOOS pain in the N and C groups were performed using the Mann–Whitney *U* test. The association between KOOS pain and CS was examined using a multiple linear regression model, which included covariates (age, sex, BMI, KL grade, PSEQ, and BS-POP) and scaled estimated regression coefficients (*β*). The variance inflation factor (VIF) is a measure of multicollinearity in a set of multiple regression variables, and a high VIF indicates that the associated independent variable is highly collinear with other variables in the model. According to a previous study, the difference in KOOS pain in KOA individuals between the two groups with and without CS was 15.5, with a common standard deviation of 11.12% [22]. From this, an effect size of 1.39 was estimated. The sample size calculation was performed using G*Power 3.1.9.7 [23]. Assuming a Mann–Whitney *U*-test between the two groups with and without CS with an effect size of 1.39, a significance level of 5%, and a power of 80–90%, the minimum required sample size was calculated to be 20–26 cases. Probability (*p*)-values < 0.05 were considered significant. All analyses were conducted using JMP PRO 16 (SAS Institute, Cary, NC, USA).

## Results

### Patients’ characteristics

The characteristics of the 66 patients are summarized in Table 1. There were no significant differences in characteristics between the N and C groups.

**Table 1:** Participants’ characteristics

	All participants *n* = 66	N group *n* = 33	C group *n* = 33	*p*-value
Age, years mean (95%CI)	72.1 (70.7–73.4)	71.4 (69.7–73.2)	72.7 (70.7–74.7)	0.5226
Age, years				
< 65	7 (10.6)	3 (9.1)	4 (12.1)	0.2184
65–74	35 (53.0)	22 (66.7)	13 (39.4)	
≥ 75	24 (36.4)	8 (24.2)	16 (48.5)	
Sex				
Male	15 (22.7)	6 (18.1)	9 (27.2)	0.7690
Female	51 (77.3)	27 (81.9)	24 (72.8)	
BMI				
< 25	28 (42.4)	17 (51.5)	11 (33.3)	0.3191
≥ 25	38 (57.6)	16 (48.5)	22 (66.7)	
KL grade				
KL-3	19 (28.8)	9 (27.3)	10 (30.3)	0.7857
KL-4	47 (71.2)	24 (72.7)	23 (69.7)	
PSEQ				
< 40	41 (62.1)	17 (51.5)	24 (72.7)	0.0757
≥ 40	25 (37.9)	16 (48.5)	9 (27.3)	
BS-POP				
Normal BS-POP	50 (75.8)	28 (84.8)	22 (66.7)	0.0848
Abnormal BS-POP	16 (24.2)	5 (15.2)	11 (33.3)	

The data are presented as *n* (%)

*BMI* body mass index, *KL grade* Kellgren‒Lawrence grade, *PSEQ* Pain Self-Efficacy Questionnaire, *BS-POP* Brief Scale for Psychiatric Problems in Orthopedic Patients

### Comparative analyses of KOOS pain between the N and C groups by Mann–Whitney U test

**Fig. 1:**
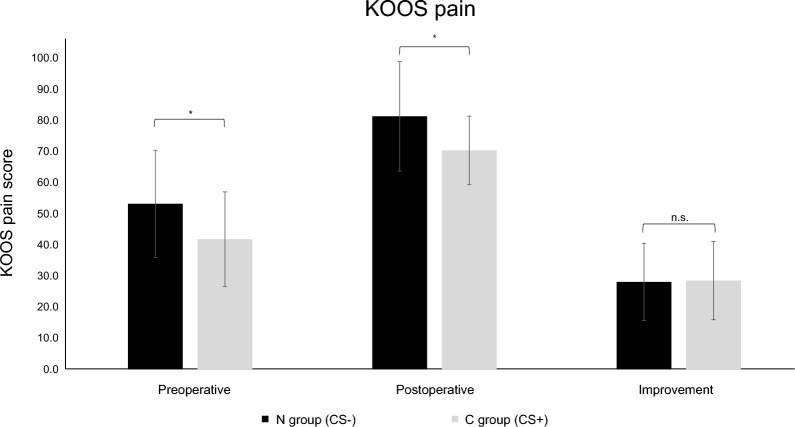
The asterisk (*) indicates (*p*)-values < 0.05. The pre- and post-operative KOOS pain scores were significantly lower in the C group compared with the N group, but there was no significant difference in improvement of KOOS pain

Table 2 shows the pre- and post-operative KOOS pain values for all patients, the N group, and the C group, along with the degree of improvement in KOOS pain scores. The pre- and post-operative KOOS pain scores were significantly lower in the C group compared with the N group. There was no significant difference in the improvement of KOOS pain between the N and C groups (Fig. 1).

**Table 2:** Comparative analyses of KOOS pain between the N and C groups by Mann–Whitney *U* test

KOOS pain	All participants *n* = 66	N group *n* = 33	C group *n* = 33	*p*-Value
Preoperative	47.5 (43.2–51.9)	53.2 (47.3–59.1)	41.8 (35.9–47.7)	0.0082
Postoperative	75.8 (72.3–79.4)	81.3 (76.7–85.9)	70.4 (65.8–75.0)	0.0014
Improvement	28.3 (23.5–33.1)	28.1 (21.2–35.0)	28.5 (21.7–35.4)	0.9283

The data are presented as mean (95% CI)

*KOOS* Knee injury and Osteoarthritis Outcome Score

### Factors that influenced preoperative KOOS pain in the multiple linear regression analysis

High CS had a negative effect on the patients’ preoperative KOOS pain scores (*β*: −0.28, 95% confidence interval [CI] −18.53, −0.92). High pain self-efficacy had a positive impact on preoperative KOOS pain (*β*: 0.25, 95%CI 0.32, 18.08), whereas age, sex, BMI, KL grade, and abnormal BS-POP did not significantly influence preoperative KOOS pain (Table 3). As the VIF of each covariate was quite low in this analysis, there was no multicollinearity between the covariates.

**Table 3:** Influence factors of preoperative KOOS pain in multiple linear regression analysis

Preoperative KOOS pain	*β*	95% CI	*p*-Value	VIF
Age, per 1 year	0.14	−0.36, 1.27	0.2660	1.12
Female	−0.18	−17.36, 2.51	0.1401	1.05
BMI ≥ 25	−0.10	−12.25, 5.76	0.4732	1.19
KL grade 4	−0.05	−11.38, 7.14	0.6485	1.06
High CS	**−0.28**	−18.53, −0.92	**0.0310**	1.17
High pain self-efficacy	**0.25**	0.32, 18.08	**0.0426**	1.12
Abnormal BS-POP	−0.06	−3.53, 10.77	0.6461	1.18

Multiple linear regression analysis was performed with age, sex, BMI, KL grade, CSI-9, PSEQ, and BS-POP

*KOOS* Knee Injury and Osteoarthritis Outcome Score, *BMI* body mass index, *KL grade* Kellgren‒Lawrence grade, *CSI-9* Central Sensitization Inventory-9, *PSEQ* Pain Self-Efficacy Questionnaire, *BS-POP* Brief Scale for Psychiatric Problems in Orthopedic Patients, *VIF* variance inflation factor

Factors with significant differences are shown in bold emphasis

### Factors that influenced postoperative KOOS pain in the multiple linear regression analysis

High CS had a negative effect on the patients’ postoperative KOOS pain (*β*: −0.26, 95%CI −14.09, −0.44) whereas age, sex, BMI, KL grade, high pain self-efficacy, and abnormal BS-POP did not significantly influence postoperative KOOS pain (Table 4). As the VIF of each covariate was also quite low in this analysis, there was no multicollinearity between the covariates.

**Table 4:** Influence factors of postoperative KOOS pain in multiple linear regression analysis

Postoperative KOOS pain	*β*	95% CI	*p*-Value	VIF
Age, per 1 year	−0.08	−0.84, 0.42	0.4991	1.12
Female	−0.05	−9.39, 6.01	0.6627	1.05
BMI ≥ 25	−0.21	−13.04, − 0.92	0.0874	1.19
KL grade (KL-4)	0.15	−2.58, 11.78	0.2047	1.06
High CS	**−0.26**	−14.09, −0.44	**0.0373**	1.17
High pain self-efficacy	0.16	−2.14, 11.63	0.1730	1.12
Abnormal BS-POP	−0.22	−15.20, 0.82	0.0774	1.18

Multiple linear regression analysis was performed with age, sex, BMI, KL grade, CSI-9, PSEQ, and BS-POP. Abbreviations are explained in the footnote of Table 3

Factors with significant differences are shown in bold emphasis

### Factors that influenced the improvement of KOOS pain in the multiple linear regression analysis

Age, sex, BMI, KL grade, high CS, high pain self-efficacy, and abnormal BS-POP did not significantly influence the improvement of KOOS pain (Table 5). There was no multicollinearity between the covariates, as the VIF of each covariate was quite low in this analysis.

**Table 5:** Influence factors of KOOS pain postoperative changes in multiple linear regression analysis

Improvement of KOOS pain	*β*	95% CI	*p*-Value	VIF
Age, per 1 year	−0.18	−1.64, 0.30	0.1704	1.12
Female	0.12	−6.07, 17.55	0.3348	1.05
BMI ≥ 25	−0.07	−13.52, 7.89	0.6007	1.19
KL grade 4	0.16	−4.29, 17.73	0.2267	1.06
High CS	0.06	−8.01, 12.93	0.6398	1.17
High pain self-efficacy	−0.11	−15.01, 6.11	0.4021	1.12
Abnormal BS-POP	−0.11	−17.10, 7.47	0.4363	1.18

Multiple linear regression analysis was performed with age, sex, BMI, KL grade, CSI-9, PSEQ, and BS-POP. Abbreviations are explained in the footnote of Table 3

## Discussion

Our findings revealed that CS had a negative impact on preoperative and postoperative knee pain in patients, but it did not affect the improvement of knee pain. In previous studies, higher levels of CS have been reported in patients with bilateral KOA compared with those with unilateral KOA [24]. This suggests that CS ratios may differ between patients with severe unilateral OA and those with severe bilateral OA. To reduce such bias, we analyzed the cases of only patients with radiographically defined severe bilateral KOA. This study is the first to analyze the impact of CS on perioperative knee pain in bilateral TKA for bilateral severe KOA. Our results corroborate and extend the prior studies’ findings that patients with preoperative CS have been reported to have worse pre- and post-operative pain and outcomes [10, 11].

The pain mechanism of KOA is explained by two factors: nociceptive pain associated with structural changes and inflammation in the joint [25], and CS pain caused by changes in the spinal cord and brain [26]. The results of this study show that TKA, with or without CS, provides sufficient pain relief for severe KOA. These results suggest that a TKA improves nociceptive pain. However, CS-derived pain exacerbates preoperative and postoperative knee pain, which is not improved by TKA. In other words, a TKA is effective enough for nociceptive pain associated with KOA, but not for pain derived from CS, where there is no clear nociception. In patients with CS in addition to severe KOA, it is important to improve CS-derived pain preoperatively. Exercise therapy, cognitive behavioral therapy, and medications such as duloxetine reduce CS-derived knee pain [27, 28]. This can be a key factor in the treatment of persistent pain after TKA surgery. Further research is required to investigate this issue.

We included pain self-efficacy and depression/anxiety as potential confounders in the present analyses to reveal the impact of CS on perioperative knee pain, and the results demonstrated that pain self-efficacy had a positive effect on preoperative knee pain. Our findings are consistent with the fact that pain self-efficacy is a protective factor that promotes adaptation even in painful situations [16]. In contrast, we observed that depression/anxiety had no significant effect on perioperative knee pain. This result differs from that of a previous study [27], but this may be explained by the coexistence of CS and psychological factors. Cases involving CS often include psychological conditions such as depression/anxiety and social characteristics such as interpersonal relationship disorders [29]. The relationship between CS and depression/anxiety has been inconsistent in previous studies, and the study populations were limited to specific pain-related diseases [30–32]. Continued research on pain-related diseases and the accumulation of more knowledge in this area are necessary. It is important to consider the possibility of coexisting CS in patients with psychological factors such as depression/anxiety.

Several study limitations must be addressed. First, because the multivariate results were obtained for a cross-sectional analysis at each time point, a causal relationship could not be determined. Second, we did not investigate the patients’ detailed history of treatment for KOA or the duration of their disease, which might have affected their knee pain. Third, the sample size (66 patients) was small; however, we included only patients with severe bilateral KOA as defined by plane radiographs in order to reduce the bias caused by the differences in the level of CS between patients with bilateral KOA and those with unilateral KOA [24]. Fourth, our primary analysis did not compare overall clinical outcome scores and did not include other pain assessment tools such as a pain visual analog scale (VAS). However, the study’s focus was on pain and attempted a more objective assessment of pain. Finally, although this study measured the short-term results at 3 months after TKA surgery, a longer follow-up period may have had a significant impact on the results of this study [33]; therefore, longer-term follow-up is also necessary.

## Conclusions

The results of our analyses demonstrated that central sensitization had a negative impact on preoperative and postoperative knee pain in patients undergoing bilateral TKA. However, CS did not affect the improvement of knee pain. TKA provides sufficient pain relief for severe KOA, with or without CS. Further research is required to improve pre- and post-operative knee pain in KOA patients with CS.

## Data Availability

The datasets generated during and/or analyzed during this study are available from the corresponding author on reasonable request.

## Abbreviations

KOA: Knee osteoarthritis
TKA: Total knee arthroplasty
CPSP: Chronic postsurgical pain
CS: Central sensitization
KOOS: Knee Injury and Osteoarthritis Outcome Score
KL grade: Kellgren‒Lawrence grade
BMI: Body mass index
CSI-9: Central Sensitization Inventory-9
PSEQ: Pain Self-Efficacy Questionnaire
BS-POP: Brief Scale for Psychiatric Problems in Orthopedic Patients
CSI: Central Sensitization Inventory
VIF: Variance inflation factor
